# Association of Body Mass Index With Outcomes Among Patients With Head and Neck Cancer Treated With Chemoradiotherapy

**DOI:** 10.1001/jamanetworkopen.2023.20513

**Published:** 2023-06-27

**Authors:** Sung Jun Ma, Michael Khan, Udit Chatterjee, Sharon Santhosh, Mahnoor Hashmi, Jasmin Gill, Brian Yu, Austin Iovoli, Mark Farrugia, Kimberly Wooten, Vishal Gupta, Ryan McSpadden, Han Yu, Moni A. Kuriakose, Michael R. Markiewicz, Ayham Al-Afif, Wesley L. Hicks, Mukund Seshadri, Andrew D. Ray, Elizabeth Repasky, Anurag K. Singh

**Affiliations:** 1 Department of Radiation Medicine, Roswell Park Comprehensive Cancer Center, Buffalo, New York; 2 Jacobs School of Medicine and Biomedical Sciences, University at Buffalo, The State University of New York, Buffalo; 3 Dow International Medical College, Karachi, Pakistan; 4 University at Buffalo, The State University of New York, Buffalo; 5 Department of Head and Neck Surgery, Roswell Park Comprehensive Cancer Center, Buffalo, New York; 6 Department of Biostatistics and Bioinformatics, Roswell Park Comprehensive Cancer Center, Buffalo, New York; 7 Department of Oral and Maxillofacial Surgery, School of Dental Medicine, University at Buffalo, The State University of New York, Buffalo; 8 Department of Neurosurgery, Jacobs School of Medicine and Biomedical Sciences, University at Buffalo, The State University of New York, Buffalo; 9 Department of Surgery, Jacobs School of Medicine and Biomedical Sciences, University at Buffalo, The State University of New York, Buffalo; 10 Department of Oral Oncology, Roswell Park Comprehensive Cancer Center, Buffalo, New York; 11 Department of Cancer Prevention and Control, Roswell Park Comprehensive Cancer Center, Buffalo, New York; 12 Department of Immunology, Roswell Park Comprehensive Cancer Center, Buffalo, New York

## Abstract

**Importance:**

Combined modality therapy, such as chemoradiotherapy, often results in significant morbidity among patients with head and neck cancer. Although the role of body mass index (BMI) varies based on cancer subtypes, its association with treatment response, tumor recurrence, and survival outcomes among patients with head and neck cancer remains unclear.

**Objective:**

To evaluate the role of BMI in treatment response, tumor recurrence, and survival outcomes among patients with head and neck cancer undergoing chemoradiotherapy.

**Design, Setting, and Participants:**

This retrospective, observational, single-institution cohort study conducted at a comprehensive cancer center included 445 patients with nonmetastatic head and neck cancer who underwent chemoradiotherapy from January 1, 2005, to January 31, 2021.

**Exposure:**

Normal vs overweight or obese BMI.

**Main Outcomes and Measures:**

Metabolic response after chemoradiotherapy, locoregional failure (LRF), distant failure (DF), overall survival (OS), and progression-free survival (PFS), with Bonferroni correction used to adjust for multiple comparisons and *P* < .025 being considered statistically significant.

**Results:**

A total of 445 patients (373 men [83.8%]; median age, 61 years [IQR, 55-66 years]; 107 [24.0%] with normal BMI, 179 [40.2%] with overweight BMI, and 159 [35.7%] with obese BMI) were included for analysis. Median follow-up was 48.1 months (IQR, 24.7-74.9 months). On Cox proportional hazards regression multivariable analysis, only overweight BMI was associated with improved OS (5-year OS, 71.5% vs 58.4%; adjusted hazard ratio [AHR], 0.59 [95% CI, 0.39-0.91]; *P* = .02) and PFS (5-year PFS, 68.3% vs 50.8%; AHR, 0.51 [95% CI, 0.34-0.75]; *P* < .001). On logistic multivariable analysis, overweight BMI (91.6% vs 73.8%; adjusted odds ratio [AOR], 0.86 [95% CI, 0.80-0.93]; *P* < .001) and obese BMI (90.6% vs 73.8%; AOR, 0.89 [95% CI, 0.81-0.96]; *P* = .005) were associated with complete metabolic response on follow-up positron emission tomography–computed tomography after treatments. On Fine-Gray multivariable analysis, overweight BMI was associated with reduction in LRF (5-year LRF, 7.0% vs 25.9%; AHR, 0.30 [95% CI, 0.12-0.71]; *P* = .01), but not DF (5-year DF, 17.4% vs 21.5%; AHR, 0.92 [95% CI, 0.47-1.77]; *P* = .79). Obese BMI was not associated with LRF (5-year LRF, 10.4% vs 25.9%; AHR, 0.63 [95% CI, 0.29-1.37]; *P* = .24) or DF (5-year DF, 15.0% vs 21.5%; AHR, 0.70 [95% CI, 0.35-1.38]; *P* = .30).

**Conclusion:**

In this cohort study of patients with head and neck cancer, when compared with normal BMI, overweight BMI was an independent factor favorably associated with complete response after treatments, OS, PFS, and LRF. Further investigations are warranted to improve understanding on the role of BMI among patients with head and neck cancer.

## Key Points

**Question:**

What is the association of overweight and obese body mass index (BMI) with posttreatment response, tumor recurrence, and survival outcomes among patients with head and neck cancer who underwent chemoradiotherapy?

**Findings:**

In this cohort study involving 445 patients, both overweight and obese BMI were associated with complete metabolic response after chemoradiotherapy. Only overweight BMI was associated with improved overall survival, progression-free survival, and reduction in locoregional failure.

**Meaning:**

This study suggests that overweight BMI is an independent factor favorably associated with complete metabolic response after chemoradiotherapy, survival, and locoregional failure.

## Teaser

This cohort study evaluates the role of body mass index (BMI) in treatment response, tumor recurrence, and survival outcomes among patients with head and neck cancer undergoing chemoradiotherapy.

## Introduction

The prevalence of obesity is anticipated to increase, with nearly 1 in 2 adults having obesity by 2030.^1^ The prognostic role of body mass index (BMI; calculated as weight in kilograms divided by height in meters squared) may vary based on cancer subtypes.^2^

Previous meta-analysis and prospective studies showed that both obesity and overweight were associated with worse all-cause mortality,^3^ cancer-related mortality,^4^ and the incidence of multiple types of cancer.^5^ However, while similar findings were noted for breast, ovarian, and colorectal cancer,^6,7,8^ obesity was a favorable prognostic factor for survival in lung cancer^2,9,10^ and renal cell carcinoma.^2,11^

A correlation between BMI and survival among patients with head and neck cancer was not observed in a meta-analysis.^2^ Combined modality therapies, such as chemoradiotherapy, often result in weight loss with muscle mass depletion, which is associated with poor prognosis.^12,13,14,15^ The role of BMI in this setting remains unclear. To address these knowledge gaps, we performed an observational cohort study to evaluate the association between BMI and survival outcomes.

## Methods

Our study was performed under a protocol approved by the Roswell Park Comprehensive Cancer Center institutional review board. The Strengthening the Reporting of Observational Studies in Epidemiology (STROBE) reporting guideline was reviewed, and our study follows the guideline. The study was conducted in accordance with the Declaration of Helsinki.^16^ A waiver of consent was obtained from the institutional review board of the Roswell Park Comprehensive Cancer Center due to the retrospective nature of the study making consent impractical and because contacting patients to obtain consent would pose a greater risk than the waiver.

Our retrospective database was queried for patients with head and neck cancer who underwent curative-intent definitive chemoradiotherapy at the Roswell Park Comprehensive Cancer Center between January 1, 2005, and January 31, 2021. Patients were excluded if they underwent surgery or radiotherapy alone, received a diagnosis of metastatic cancer, or had unknown BMI. Patients with low BMI (underweight, <18.5) were also excluded due to a small sample size (n <15).

Body mass index is stratified by normal weight (18.5-24.9), overweight (25.0-29.9), and obese (≥30). Other variables of interest were extracted, including age, self-reported gender, smoking history, Karnofsky performance status, race and ethnicity, number of comorbidities, primary disease site, cancer staging based on the *American Joint Committee on Cancer Staging Manual*, 7th edition,^17^ human papillomavirus (HPV) status, and chemotherapy. All missing values were coded as unknown for analysis. Other clinically pertinent variables were not captured in the database, such as treatment-related toxic effects. Race and ethnicity were self-reported, and this information was extracted from the electronic health record. Among patients who self-reported other racial and ethnic backgrounds, they included African American, American Indian or Alaska Native, Asian, Hispanic, and those who were unknown or declined to answer. Such categories were combined as a single group prior to performing our analyses because it would be challenging to show meaningful differences in outcomes due to their small subgroup sample sizes.

The primary end points of our study were overall survival (OS) and progression-free survival (PFS). These outcomes were defined as the time intervals from diagnosis to any death or last follow-up and from diagnosis to tumor progression or any death or last follow-up, respectively. Other end points included metabolic response of disease on positron emission tomography–computed tomography (PET-CT) after completing radiotherapy, locoregional failure (LRF), distant failure (DF), and treatment interruptions, defined as more than 56 days of the radiotherapy treatment course.^18,19,20^

### Statistical Analysis

Comparison of baseline characteristics was performed using the Fisher exact test and the Mann-Whitney test as appropriate. Evaluation of survival outcomes was performed using the Kaplan-Meier method, log-rank tests, and Cox proportional hazards regression multivariable analyses. Logistic multivariable analysis was performed to identify variables associated with posttreatment responses and treatment interruptions. Fine-Gray multivariable analysis was performed to evaluate LRF and DF outcomes with death as a competing event. All multivariable analysis models were constructed using all patient and tumor variables as listed previously.

Propensity score matching was used to reduce selection bias. All baseline characteristics were considered for matching as deemed clinically pertinent. Matching was performed based on the nearest neighbor method in a 1:1 ratio with no replacements and a caliper distance of 0.2.^21^ Subgroup analyses were also performed to evaluate OS, PFS, LRF, and DF outcomes based on HPV status, which was assessed using p16 status among patients with oropharyngeal cancer.

Bonferroni correction was used to adjust for multiple comparison (normal vs overweight BMI and normal vs obese BMI). All *P* values were 2-sided, and *P* < .025 was deemed statistically significant. All statistical analyses were performed using R, version 4.2.1 (R Group for Statistical Computing).

## Results

A total of 445 patients (373 men [83.8%]; median age, 61 years [IQR, 55-66 years]; 107 [24.0%] with normal BMI, 179 [40.2%] with overweight BMI, and 159 [35.7%] with obese BMI) met our criteria (Table). Most patients had a good Karnofsky performance status of 90 to 100 (339 [76.2%]) and underwent definitive chemoradiotherapy for oropharyngeal cancer (262 [58.9%]). There were 3 patients (0.7%) with treatment interruptions. Median follow-up was 48.1 months (IQR, 24.7-74.9 months).

**Table.** Baseline Characteristics

Characteristic	Before matching, No. (%) (N = 445)	After matching					
		No. (%)		*P* value	No. (%)		*P* value
		Normal BMI (n = 81)	Overweight BMI (n = 81)		Normal BMI (n = 65)	Obese BMI (n = 65)	
BMI							
Normal (18.5-24.9)	107 (24.0)	81 (100.0)	0	NA	65 (100.0)	0	NA
Overweight (25.0-29.9)	179 (40.2)	0	81 (100.0)		0	0	
Obese (≥30)	159 (35.7)	0	0		0	65 (100.0)	
Gender							
Man	373 (83.8)	67 (82.7)	66 (81.5)	>.99	54 (83.1)	55 (84.6)	>.99
Woman	72 (16.2)	14 (17.3)	15 (18.5)		11 (16.9)	10 (15.4)	
Smoker							
Never or former	369 (82.9)	64 (79.0)	63 (77.8)	>.99	55 (84.6)	51 (78.5)	.50
Current	76 (17.1)	17 (21.0)	18 (22.2)		10 (15.4)	14 (21.5)	
Age, y							
<65	320 (71.9)	59 (72.8)	61 (75.3)	.86	45 (69.2)	50 (76.9)	.43
≥65	125 (28.1)	22 (27.2)	20 (24.7)		20 (30.8)	15 (23.1)	
KPS							
<90	106 (23.8)	22 (27.2)	22 (27.2)	>.99	16 (24.6)	16 (24.6)	>.99
90-100	339 (76.2)	59 (72.8)	59 (72.8)		49 (75.4)	49 (75.4)	
Race and ethnicity							
White	390 (87.6)	72 (88.9)	67 (82.7)	.37	55 (84.6)	56 (86.2)	>.99
Other^a^	55 (12.4)	9 (11.1)	14 (17.3)		10 (15.4)	9 (13.8)	
No. of comorbidities							
0	71 (16.0)	12 (14.8)	15 (18.5)	.78	11 (16.9)	9 (13.8)	.88
1-3	272 (61.1)	51 (63.0)	47 (58.0)		39 (60.0)	42 (64.6)	
>3	102 (22.9)	18 (22.2)	19 (23.5)		15 (23.1)	14 (21.5)	
Primary disease site							
Oropharynx	262 (58.9)	42 (51.9)	45 (55.6)	.91	35 (53.8)	38 (58.5)	.69
Larynx	102 (22.9)	24 (29.6)	23 (28.4)		18 (27.7)	19 (29.2)	
Other	81 (18.2)	15 (18.5)	13 (16.0)		12 (18.5)	8 (12.3)	
T staging							
1-2	231 (51.9)	30 (37.0)	36 (44.4)	.42	27 (41.5)	30 (46.2)	.72
3-4	214 (48.1)	51 (63.0)	45 (55.6)		38 (58.5)	35 (53.8)	
N staging							
0	87 (19.6)	18 (22.2)	20 (24.7)	.81	15 (23.1)	15 (23.1)	>.99
1	44 (9.9)	13 (16.0)	9 (11.1)		6 (9.2)	6 (9.2)	
2	278 (62.5)	44 (54.3)	47 (58.0)		40 (61.5)	39 (60.0)	
3	36 (8.1)	6 (7.4)	5 (6.2)		4 (6.2)	5 (7.7)	
HPV							
Negative	76 (17.1)	14 (17.3)	14 (17.3)	>.99	12 (18.5)	8 (12.3)	.68
Positive	225 (50.6)	31 (38.3)	32 (39.5)		29 (44.6)	31 (47.7)	
Not available	144 (32.4)	36 (44.4)	35 (43.2)		24 (36.9)	26 (40.0)	
Chemotherapy							
Cisplatin	374 (84.0)	70 (86.4)	70 (86.4)	>.99	54 (83.1)	56 (86.2)	.81
Other	71 (16.0)	11 (13.6)	11 (13.6)		11 (16.9)	9 (13.8)	

Abbreviations: BMI, body mass index (calculated as weight in kilograms divided by height in meters squared); HPV, human papillomavirus; KPS, Karnofsky performance status; NA, not available.

^a^ Among patients who self-reported other racial and ethnic backgrounds, they included African American, American Indian or Alaska Native, Asian, Hispanic, and those who were unknown or declined to answer.

On Cox proportional hazards regression multivariable analysis (eTable 1 in Supplement 1), overweight BMI was associated with improved OS (5-year OS, 71.5% vs 58.4%; adjusted hazard ratio [AHR], 0.59 [95% CI, 0.39-0.91]; *P* = .02) and PFS (5-year PFS, 68.3% vs 50.8%; AHR, 0.51 [95% CI, 0.34-0.75]; *P* < .001). Obese BMI was not associated with either OS (AHR, 0.62 [95% CI, 0.39-0.98]; *P* = .04) or PFS (AHR, 0.66 [95% CI, 0.44-0.99]; *P* = .04). On logistic multivariable analysis (eTable 2 in Supplement 1), having overweight BMI (91.6% vs 73.8%; adjusted odds ratio [AOR], 0.86 [95% CI, 0.80-0.93]; *P* < .001) and obese BMI (90.6% vs 73.8%; AOR, 0.89 [95% CI, 0.81-0.96]; *P* = .005) were associated with complete metabolic response on follow-up PET-CT after treatments. Given the small number of treatment interruptions seen in our cohort, there was no association between BMI and treatment interruptions (overweight BMI: AOR, 1.00 [95% CI, 0.98-1.03]; *P* = .69; obese BMI: AOR, 0.99 [95% CI, 0.97-1.01]; *P* = .43).

On Fine-Gray multivariable analysis (eTable 3 in Supplement 1), overweight BMI was associated with a reduction in LRF (5-year LRF, 7.0% vs 25.9%; AHR, 0.30 [95% CI, 0.12-0.71]; *P* = .01) but not DF (5-year DF, 17.4% vs 21.5%; AHR, 0.92 [95% CI, 0.47-1.77]; *P* = .79). Obese BMI was not associated with LRF (5-year LRF, 10.4% vs 25.9%; AHR, 0.63 [95% CI, 0.29-1.37]; *P* = .24) or DF (5-year DF, 15.0% vs 21.5%; AHR, 0.70 [95% CI, 0.35-1.38]; *P* = .30).

**Figure 1.**
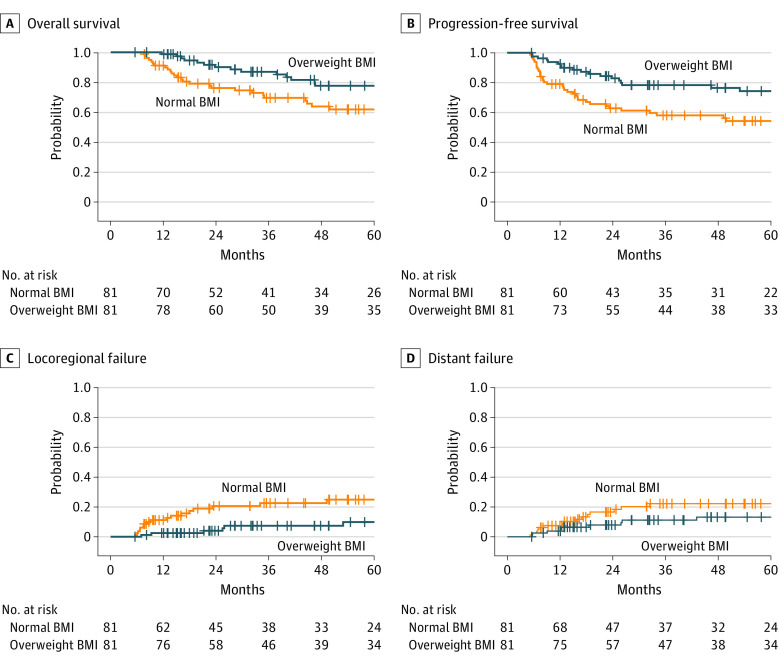
Kaplan-Meier and Cumulative Incidence Curves for Overall Survival, Progression-Free Survival, Locoregional Failure, and Distant Failure for Overweight vs Normal Body Mass Index (BMI) After Propensity Score Matching

**Figure 2.**
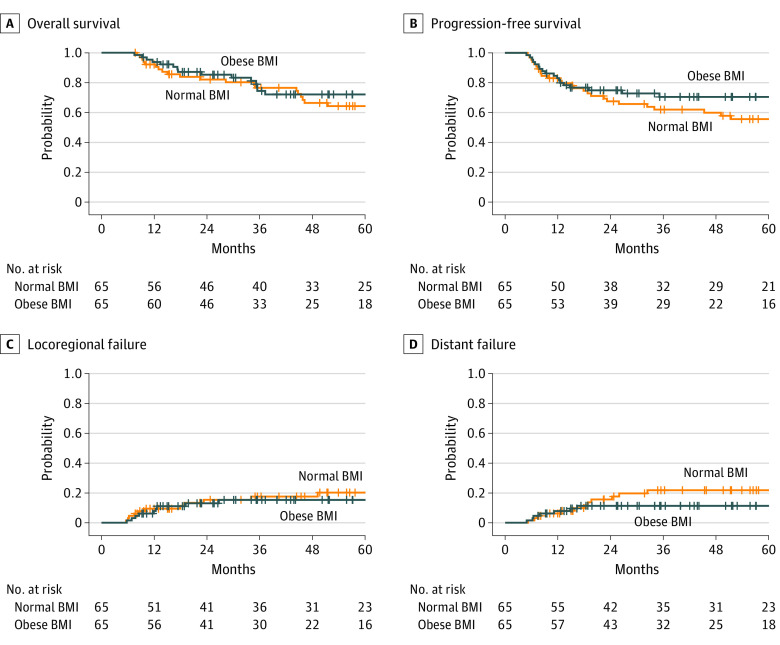
Kaplan-Meier and Cumulative Incidence Curves for Overall Survival, Progression-Free Survival, Locoregional Failure, and Distant Failure for Obese vs Normal Body Mass Index (BMI) After Propensity Score Matching

After propensity score matching, 81 matched pairs were identified for normal vs overweight BMI, and 65 matched pairs were identified for normal vs obese BMI. All baseline characteristics were well balanced (Table). Outcomes similar to the multivariable analysis were observed for overweight BMI (OS: AHR, 0.43 [95% CI, 0.24-0.77]; *P* = .004; PFS: AHR, 0.42 [95% CI, 0.25-0.71]; *P* = .001; LRF: AHR, 0.35 [95% CI, 0.15-0.82]; *P* = .02; DF: AHR, 0.66 [95% CI, 0.30-1.47]; *P* = .31) (Figure 1) and obese BMI (OS: AHR, 0.66 [95% CI, 0.36-1.22]; *P* = .18; PFS: AHR, 0.61 [95% CI, 0.35-1.08]; *P* = .09; LRF: AHR, 0.77 [95% CI, 0.33-1.78]; *P* = .53; DF: AHR, 0.59 [95% CI, 0.23-1.48]; *P* = .26) (Figure 2).

On subgroup analysis, 76 patients with oropharyngeal cancer were identified for p16-negative, and 225 patients with oropharyngeal cancer were identified for p16-positive cases. When Cox and Fine-Gray multivariable analyses were repeated, the only statistically significant outcome was LRF for those with overweight BMI (AHR, 0.02 [95% CI, 0.001-0.29]; *P* = .005) (eTable 4 in Supplement 1).

## Discussion

To our knowledge, this is the largest study involving patients in the US treated with chemoradiotherapy for head and neck cancer that evaluated the role of BMI as a factor associated with survival, treatment response, and tumor recurrence outcomes. Overweight BMI and obese BMI were associated with complete metabolic response on follow-up PET-CT; however, only overweight BMI was an independent factor favorably associated with improved OS and PFS and a reduction in LRF. No association for OS and BMI was observed among HPV-positive patients.

Our finding of an association of overweight BMI with improved survival is consistent with a growing body of literature suggesting a higher BMI as a favorable prognostic factor.^22,23,24,25,26,27,28,29^ However, obese BMI was not associated with OS in our study. This finding is consistent with several reports,^30,31,32^ whereas other studies have reported survival benefits associated with obese BMI.^22,23,24,25,28,29^ Such discrepancies may be due to a nonlinear association between BMI and survival, with the highest survival seen in the overweight BMI range.^33,34^

To our knowledge, this is the first report for head and neck cancer to show that overweight BMI and obese BMI are associated with complete metabolic response on follow-up PET-CT. Our finding is consistent with another study suggesting a higher likelihood of pathologic complete response in rectal cancer among patients with obese BMI,^35^ whereas it is inconsistent with other studies suggesting that obese BMI is adversely associated with pathologic complete response in breast and rectal cancers.^36,37,38,39^

Reasons for this complex association may be multifactorial. Although obese BMI has been associated with worse postoperative complications,^31,40^ chronic inflammation for tumor development,^41^ and reduced antitumor immune response,^42^ several studies have suggested that obese BMI is a nutrient reserve to overcome toxic effects from combined modality therapies,^33^ which may be associated with improved LRF^24^ and DF.^25,26^ Such a complex interplay may explain the conflicting association between treatment-associated weight loss and survival for patients with head and neck cancer.^15,27,43,44^ This interplay may also explain the variations seen in markers for systematic inflammation,^45^ such as the neutrophil-lymphocyte ratio.^46^ Further complicating matters, studies have suggested that BMI alone may not be representative of one’s body fat composition and cachexia.^47,48^ Another quantitative measure correlated with BMI is skeletal muscle depletion measured based on CT imaging, which has been shown to be associated with worse survival^24,49^ and quality of life^50^ among patients with head and neck cancer.

In our study, BMI was not associated with survival outcomes among HPV-positive patients, consistent with prior studies.^32,51^ Although a few other studies have suggested that a higher BMI is associated with improved survival,^41^ they also included patients with an underweight BMI as a reference group, which was previously shown to be associated with worse survival outcomes.^33,34^ Although a lack of association between BMI, HPV, and survival in our study may be due to smaller subgroup sample sizes, interaction among these variables warrants further investigation. For example, despite adipose tissue–promoting pathways, including PI3K-PTEN-Akt-mTOR and Ras-Raf-MAPK associated with HPV-associated head and neck cancers,^52^ patients with a high BMI were more likely to have greater treatment-related weight loss^44,53^ associated with changes in tumor microenvironment and inflammation that may potentiate treatments.^54^

### Limitations

Our retrospective study has inherent limitations. In our study, BMI was analyzed as a categorical variable with 3 different strata (normal, overweight, and obese) instead of as a continuous variable. The association between BMI and survival outcomes has been previously shown to be complex and nonlinear,^33,34^ and there may be more clinically pertinent, model-derived BMI cutoffs associated with clinical outcomes. Our BMI variable was also collected at a single time point, and our analysis did not include dynamic changes in BMI prior to the diagnosis of head and neck cancer, during chemoradiotherapy, or after the completion of all treatments. Such changes may be more clinically pertinent in prognosticating clinical outcomes than a single measure of BMI. In addition, only 40% to 45% of patients with overweight BMI and obese BMI were matched, suggesting that our matched cohort may not be representative of our overall cohort. However, our findings from the matched cohorts were consistent with those from the overall cohort. Other clinical outcomes, such as toxicity profiles, were unavailable for analysis. Furthermore, our findings may not be generalizable for other patient cohorts who underwent surgery, induction systemic therapy, or radiotherapy alone.

## Conclusions

Our cohort study suggests that overweight BMI is an independent, favorable factor associated with complete response after treatments, OS, PFS, and LRF. Further investigations are warranted to improve our understanding on the role of BMI among patients with head and neck cancer.

## Notes

Supplement 1. **eTable 1.** Cox Multivariable Analysis for Overall and Progression-Free Survival **eTable 2.** Logistic Multivariable Analysis for Post-treatment Response **eTable 3.** Fine-Gray Multivariable Analysis for Locoregional and Distant Failure **eTable 4.** Cox and Fine-Gray Multivariable Analysis for Survival and Tumor Recurrence Outcomes Stratified by p16 Status

Supplement 2.
Data Sharing Statement
