# Supplementary material for: Association of Body Mass Index With Outcomes Among Patients With Head and Neck Cancer Treated With Chemoradiotherapy
Source: JAMA Netw Open. 2023 Jun 27;6(6):e2320513. doi: 10.1001/jamanetworkopen.2023.20513 (PMC10300672; doi:10.1001/jamanetworkopen.2023.20513)
Supplement: Supplement 1. — eTable 1. Cox Multivariable Analysis for Overall and Progression-Free Survival eTable 2. Logistic Multivariable Analysis for Post-treatment Response eTable 3. Fine-Gray Multivariable Analysis for Locoregional and Distant Failure eTable 4. Cox and Fine-Gray Multivariable Analysis for Survival and Tumor Recurrence Outcomes Stratified by p16 Status [file jamanetwopen-e2320513-s001.pdf]

Supplementary Online Content

Ma SJ, Khan M, Chatterjee U, et al. Association of body mass index with outcomes among patients with head and neck cancer treated with chemoradiotherapy. *JAMA Netw Open.* 2023;6(6):e2320513. doi:10.1001/jamanetworkopen.2023.20513

eTable 1. Cox Multivariable Analysis for Overall and Progression-Free Survival
eTable 2. Logistic Multivariable Analysis for Post-treatment Response
eTable 3. Fine-Gray Multivariable Analysis for Locoregional and Distant Failure
eTable 4. Cox and Fine-Gray Multivariable Analysis for Survival and Tumor Recurrence Outcomes Stratified by p16 Status

This supplementary material has been provided by the authors to give readers additional information about their work.

© 2023 Ma SJ et al. *JAMA Network Open.*

eTable 1. Cox Multivariable Analysis for Overall and Progression-Free Survival

	Overall survival			Progression-free survival		
	aHR	95% CI	P	aHR	95% CI	P
BMI						
Normal	Reference			Reference		
Overweight	0.59	0.39-0.91	0.02	0.51	0.34-0.75	<0.001
Obese	0.62	0.39-0.98	0.04	0.66	0.44-0.99	0.04
Gender						
Male	Reference			Reference		
Female	0.64	0.38-1.07	0.09	0.75	0.48-1.17	0.2
Smoker						
Never/Former	Reference			Reference		
Current	1.5	0.98-2.31	0.06	1.47	1.00-2.18	0.05
Age						
<65	Reference			Reference		
65 or older	1.71	1.16-2.52	0.007	1.58	1.12-2.25	0.01
KPS						
<90	Reference			Reference		
90-100	0.65	0.44-0.96	0.03	0.72	0.51-1.03	0.08
Race						
White	Reference			Reference		
Other	1.22	0.73-2.04	0.45	0.88	0.54-1.44	0.61
Comorbidity						
0	Reference			Reference		
1-3	0.62	0.36-1.04	0.07	0.61	0.39-0.97	0.04
>3	1.08	0.60-1.94	0.79	0.89	0.53-1.50	0.66
Site						
Oropharynx	Reference			Reference		
Larynx	1.41	0.82-2.40	0.21	1.35	0.82-2.20	0.23
Other	1.19	0.71-1.99	0.5	1.19	0.75-1.88	0.46

© 2023 Ma SJ et al. *JAMA Network Open.*

	Overall survival			Progression-free survival		
	aHR	95% CI	P	aHR	95% CI	P
T staging						
1-2	Reference			Reference		
3-4	2.52	1.71-3.73	<0.001	2.04	1.45-2.88	<0.001
N staging						
0	Reference			Reference		
1	1.84	0.91-3.72	0.09	1.49	0.78-2.87	0.23
2	1.97	1.13-3.45	0.02	1.8	1.08-3.01	0.03
3	4.24	2.06-8.74	<0.001	5.71	3.07-10.63	<0.001
HPV						
Negative	Reference			Reference		
Positive	0.75	0.44-1.28	0.29	0.76	0.47-1.23	0.26
Not available	0.87	0.55-1.36	0.54	0.79	0.52-1.20	0.26
Chemotherapy						
Cisplatin	Reference			Reference		
Other	1.69	1.04-2.74	0.03	1.84	1.21-2.80	0.005

aHR: adjusted hazards ratio; CI: confidence interval; BMI: body mass index; KPS: Karnofsky performance status; HPV: human papillomavirus

© 2023 Ma SJ et al. *JAMA Network Open.*

eTable 2. Logistic Multivariable Analysis for Post-treatment Response

	aOR	95% CI	P
BMI			
Normal	Reference		
Overweight	0.86	0.80-0.93	<0.001
Obese	0.89	0.81-0.96	0.005
Gender			
Male	Reference		
Female	0.96	0.88-1.04	0.33
Smoker			
Never/Former	Reference		
Current	0.97	0.89-1.05	0.4
Age			
<65	Reference		
65 or older	0.94	0.88-1.01	0.1
KPS			
<90	Reference		
90-100	0.89	0.83-0.96	0.002
Race			
White	Reference		
Other	1.02	0.93-1.11	0.74
Comorbidity			
0	Reference		
1-3	0.92	0.84-1.00	0.05
>3	0.92	0.83-1.02	0.12

© 2023 Ma SJ et al. *JAMA Network Open.*

	aOR	95% CI	P
Site			
Oropharynx	Reference		
Larynx	1.04	0.94-1.15	0.45
Other	1.05	0.97-1.15	0.24
T staging			
1-2	Reference		
3-4	1.12	1.04-1.19	0.001
N staging			
0	Reference		
1	1.05	0.93-1.19	0.43
2	1.14	1.03-1.26	0.009
3	1.28	1.12-1.47	<0.001
HPV			
Negative	Reference		
Positive	0.9	0.82-0.99	0.04
Not available	0.94	0.86-1.02	0.14
Chemotherapy			
Cisplatin	Reference		
Other	1.23	1.13-1.35	<0.001

aOR: adjusted odds ratio; CI: confidence interval; BMI: body mass index; KPS: Karnofsky performance status; HPV: human papillomavirus

© 2023 Ma SJ et al. *JAMA Network Open.*

eTable 3. Fine-Gray Multivariable Analysis for Locoregional and Distant Failure

	Locoregional failure			Distant failure		
	aHR	95% CI	P	aHR	95% CI	P
BMI						
Normal	Reference			Reference		
Overweight	0.3	0.12-0.71	0.01	0.92	0.47-1.77	0.79
Obese	0.63	0.29-1.37	0.24	0.7	0.35-1.38	0.3
Gender						
Male	Reference			Reference		
Female	0.81	0.35-1.86	0.61	0.81	0.40-1.64	0.56
Smoker						
Never/Former	Reference			Reference		
Current	1.28	0.63-2.62	0.5	1.12	0.58-2.13	0.74
Age						
<65	Reference			Reference		
65 or older	1.45	0.72-2.93	0.3	0.91	0.53-1.59	0.75
KPS						
<90	Reference			Reference		
90-100	0.86	0.44-1.66	0.64	0.71	0.39-1.29	0.26
Race						
White	Reference			Reference		
Other	1.22	0.47-3.17	0.68	0.7	0.29-1.73	0.44
Comorbidity						
0	Reference			Reference		
1-3	0.69	0.31-1.51	0.35	0.8	0.36-1.78	0.59
>3	0.45	0.15-1.39	0.16	1.05	0.44-2.51	0.91
Site						
Oropharynx	Reference			Reference		
Larynx	2.04	0.65-6.46	0.23	1.52	0.72-3.21	0.28
Other	1.85	0.73-4.67	0.2	1.26	0.66-2.43	0.49

© 2023 Ma SJ et al. *JAMA Network Open.*

	Locoregional failure			Distant failure		
	aHR	95% CI	P	aHR	95% CI	P
T staging						
1-2	Reference			Reference		
3-4	2.4	1.24-4.66	0.01	2.38	1.36-4.15	0.002
N staging						
0	Reference			Reference		
1	0.83	0.24-2.81	0.76	2.08	0.53-8.08	0.29
2	0.9	0.33-2.45	0.84	5.19	1.86-14.47	0.002
3	3.86	1.16-12.87	0.03	11.16	3.45-36.16	<0.001
HPV						
Negative	Reference			Reference		
Positive	1.01	0.30-3.39	0.99	1.1	0.51-2.35	0.81
Not available	1.08	0.48-2.45	0.85	0.84	0.37-1.88	0.67
Chemotherapy						
Cisplatin	Reference			Reference		
Other	1.03	0.42-2.51	0.95	1.94	0.99-3.80	0.05

aHR: adjusted hazards ratio; CI: confidence interval; BMI: body mass index; KPS: Karnofsky performance status; HPV: human papillomavirus

© 2023 Ma SJ et al. *JAMA Network Open.*

eTable 4. Cox and Fine-Gray Multivariable Analysis for Survival and Tumor Recurrence Outcomes Stratified by p16 Status

p16-negative cohort												
	Overall survival			Progression-free survival			Locoregional failure			Distant failure		
	aHR	95% CI	P	aHR	95% CI	P	aHR	95% CI	P	aHR	95% CI	P
BMI												
Normal	Reference			Reference			Reference			Reference		
Overweight	1.49	0.48-4.64	0.5	0.65	0.21-1.96	0.44	0.33	0.01-9.51	0.52	7.13	0.54-94.5	0.14
Obese	1.98	0.61-6.44	0.26	0.65	0.21-2.01	0.45	0.95	0.03-33.8	0.98	0.05	0.004-0.80	0.03
p16-positive cohort												
	Overall survival			Progression-free survival			Locoregional failure			Distant failure		
	aHR	95% CI	P	aHR	95% CI	P	aHR	95% CI	P	aHR	95% CI	P
BMI												
Normal	Reference			Reference			Reference			Reference		
Overweight	0.91	0.43-1.93	0.81	0.54	0.27-1.06	0.07	0.02	0.001-0.29	0.005	0.56	0.24-1.34	0.19
Obese	0.72	0.30-1.69	0.45	0.6	0.30-1.22	0.16	0.32	0.08-1.19	0.09	0.4	0.16-1.01	0.05

aHR: adjusted hazards ratio; CI: confidence interval; BMI: body mass index

© 2023 Ma SJ et al. *JAMA Network Open.*
