# Supplementary material for: Association of Body Mass Index With Outcomes Among Patients With Head and Neck Cancer Treated With Chemoradiotherapy
Source: JAMA Netw Open. 2023 Jun 27;6(6):e2320513. doi: 10.1001/jamanetworkopen.2023.20513 (PMC10300672; doi:10.1001/jamanetworkopen.2023.20513)
Supplement: Supplement 2. — Data Sharing Statement [file jamanetwopen-e2320513-s002.pdf]

Data Sharing Statement

Ma. Association of Body Mass Index With Outcomes Among Patients With Head and Neck Cancer Treated With Chemoradiotherapy. *JAMA Netw Open*. Published June 27, 2023. doi:10.1001/jamanetworkopen.2023.20513

Data
Data available: No

Additional Information
Explanation for why data not available: The data underlying this article cannot be shared publicly for the privacy of individuals that participated in the study. The data are available from the corresponding author upon reasonable request.
